# Deep Learning–Based Classification of Epithelial–Mesenchymal Transition for Predicting Response to Therapy in Clear Cell Renal Cell Carcinoma

**DOI:** 10.3389/fonc.2021.782515

**Published:** 2022-01-24

**Authors:** Qiwei Chen, Yue Kuai, Shujing Wang, Xinqing Zhu, Hongyu Wang, Wenlong Liu, Liang Cheng, Deyong Yang

**Affiliations:** ^1^ Department of Urology, First Affiliated Hospital of Dalian Medical University, Dalian, China; ^2^ School of Information Science and Technology of Dalian Maritime University, Dalian, China; ^3^ School of Information and Communication Engineering, Dalian University of Technology, Dalian, China; ^4^ Department of Biochemistry and Molecular Biology, Dalian Medical University, Dalian, China; ^5^ Department of Pathology and Laboratory Medicine, Indiana University School of Medicine, Indianapolis, IN, United States

**Keywords:** clear cell renal cell carcinoma, epithelial-mesenchymal transition, deep learning, histopathology, immune checkpoint inhibitor

## Abstract

Epithelial–mesenchymal transition (EMT) profoundly impacts prognosis and immunotherapy of clear cell renal cell carcinoma (ccRCC). However, not every patient is tested for EMT status because this requires additional genetic studies. In this study, we developed an EMT gene signature to classify the H&E-stained slides from The Cancer Genome Atlas (TCGA) into epithelial and mesenchymal subtypes, then we trained a deep convolutional neural network to classify ccRCC which according to our EMT subtypes accurately and automatically and to further predict genomic data and prognosis. The clinical significance and multiomics analysis of the EMT signature was investigated. Patient cohorts from TCGA (n = 252) and whole slide images were used for training, testing, and validation using an algorithm to predict the EMT subtype. Our approach can robustly distinguish features predictive of the EMT subtype in H&E slides. Visualization techniques also detected EMT-associated histopathological features. Moreover, EMT subtypes were characterized by distinctive genomes, metabolic states, and immune components. Deep learning convolutional neural networks could be an extremely useful tool for predicting the EMT molecular classification of ccRCC tissue. The underlying multiomics information can be crucial in applying the appropriate and tailored targeted therapy to the patient.

## Highlights

In this study, we trained a deep convolutional neural network on hematoxylin and eosin (H&E) histology slides obtained from The Cancer Genome Atlas (TCGA) to classify their EMT status accurately and automatically and to further predict genomic data and prognosis. We revealed that Mes cluster demonstrated truncating mutations in PBRM1, and high expression of immune checkpoint molecules might lead to the immune escape of this cluster. Further we suggested that patients in the Mes subtype might respond better to ICIs combined with antiangiogenic therapy. Deep learning convolutional neural networks could be an extremely useful tool for predicting the EMT molecular classification of ccRCC tissue. The underlying multiomics information can be crucial in applying the appropriate and tailored targeted therapy to the patient.

## Introduction

Clear cell renal cell carcinomas (ccRCC) account for approximately 80% of all renal cancer cases, with approximately 3.8% of all cancers in United States (1). Metastatic ccRCC are pharmacologically managed, targeted therapy utilizing tyrosine kinase inhibitors (TKIs), anti-VEGF antibodies, mammalian target of rapamycin (mTOR), and/or immune checkpoint inhibitors are widely used in first and second line treatments, suggesting that treatment strategy is crucial for ccRCC treatment (2).

Epithelial–mesenchymal transition (EMT) is a multistep process in which epithelial cells gain a range of mesenchymal characteristics (3). EMT molecular stratification can predict whether patients respond to immunotherapy in several tumor types (4–6). Thus, we sought to develop an EMT gene signature that can predict genomic data and prognosis of patients with ccRCC.

All EMT stratification systems are based on complex molecular experiments (7, 8). Therefore, there is a tremendous medical need for simplifying procedures. One key could be the use of deep neural networks. Recently, Zhang et al. presented a comprehensive morphological analysis using computer vision methods including random decision forests and artificial neural networks to establish the correlation between cellular morphological features and EMT (9). Kather et al. predicted microsatellite instability (MSI) directly from histology in gastrointestinal cancer using convolutional neural networks (10). Nevertheless, unlike the typical MSI tumors, there are no standard histological criteria for EMT molecular subtypes in ccRCC patients. Herein, we investigated the deep learning neural network to precisely recognize the ccRCC EMT subtypes from whole-slide images of hematoxylin and eosin (H&E)–stained tissue from TCGA (The Cancer Genome Atlas). Additionally, we compared subtype comprehensive genomic, phenotypic, and clinical data.

## Materials and Methods

### RNA Expression Data and Somatic Exome Mutation Analysis

The RNA-Seq upper quartile normalized RSEM data was available for 539 ccRCC; all data is accessible *via* the NCI genome data commons and the Gene Expression Omnibus (https://gdc.cancer.gov/ and https://www.ncbi.nlm.nih.gov/geo/). GSE150404 has 60 samples with different stages. Clinicopathologic data for the corresponding patients, including gender, race, age, tumor location, histology classification, differentiation grade, tumor stage, and survival information, were also retrieved from the database. Only patients with both survival information and expression data available were included in this study. Analysis of the RNA data was split into miRNA analysis, mRNA signature analysis, and immune gene signature analysis. All subsequent analyses were performed in R open-source programming language. For differential expression analysis, RPKM values were calculated from RNaseq raw counts and upper quantile normalized. For hierarchical clustering and WGCNA, raw count data were processed and normalized using the variance stabilizing transformation algorithm implemented by the DESeq2 package.

Somatic exome sequencing data were downloaded for the 255 ccRCC. The tumors with sequencing data are accessible *via* the NCI genome data commons (https://gdc.cancer.gov/). The SMG that had been previously identified by the MutSigCV algorithm in the previous TCGA KIRC publications were used as the reference SMG.

### DNA Methylation Analysis

All data is available from TCGA data. To minimize the influence of tumor purity, we dichotomized the methylation data with a beta value cut off 0.3 and used the Ward method to cluster the distance matrix computed with the Jaccard Index. Heatmaps were generated based on row and column orders calculated as above and colored by dichotomized beta values. All methylation genes we selected with a beta value of 0.3 or more were considered evidence for epigenetic silencing and associated with poor survival.

### Development of EMT-Gene Signature in ccRCC

To find EMT-specific genes in ccRCC, gene expression data were analyzed from TCGA cohort. The EMT-related gene expression signature comprised 200 genes obtained from gene set “hallmark epithelial mesenchymal transition” in The Molecular Signatures Database (MSigDB, software. broadinstitute.org/gsea/msigdb). Genes were selected when the mRNA expression levels were either positively or negatively correlated with at least one of the well-known EMT markers: E-cadherin (CDH1), vimentin (VIM), N-cadherin (CDH2), and fibronectin 1 (FN1). Using a gene feature and its correlated genes, hierarchical clustering analysis was performed with the centered correlation coefficient as the measure of similarity (**Supplementary Table S1**). The patient clustering result divided the patients into two subtypes (mesenchymal and epithelial). We also calculated EMT scores based on previous studies and obtained comparable results.

### Image Preprocessing

All the slides were tiled in non-overlapping 256×256-pixel windows at a magnification of 10× using the openslide library. The tiles with more than 50% background (for which all the values are below 220 in the RGB color space) were considered to have insufficient information and were dropped while tiling (11). For the remaining tiles, we yielded 1150952 tiles, with an average of 3326 tiles per slide (minimum 174 tiles, maximum 9500 tiles). We used the Macenko method for color normalization to convert all images to a reference color space for all these tiles (12).

### Tumor Detection and EMT Subtype Detection

For tumor detection in ccRCC, we selected 1000 tiles of necrosis, stroma and tumor from the tiles respectively, and randomly split them into training, validation and testing set at a ratio of 7:1.5:1.5. We trained a convolutional neural network with the Inception V3 model to classify necrosis, stroma, and tumor tiles by transfer learning. This network was then used to detect all the tumor tiles generated from the whole-slide images. This process resulted in 1031666 tiles, with an average of 2982 tiles per slide (minimum 170 tiles, maximum 7930 tiles).

For EMT subtype detection, we trained another Inception v3 model using just the tumor tiles detected before. As we already obtained the EMT subtype for patients’ slides using our gene signature, we labeled the tiles with Epi or Mes according to the slides’ EMT subtype. That is, the label of tiles was the EMT subtype of the slides the tiles generated from. Due to the small number of Epi slides, training and validation were performed slide-wise using six-fold cross-validation. That is, the tiles associated with the same slide were grouped into the same fold. Because there were different numbers of Epi and Mes slides (6 Epi and 52 Mes in each fold) and different numbers of tumor tiles of each slide, there was an imbalance between these two classes. To minimize such imbalance, while training, we extracted all the Epi slides and downsampled the Mes slides at a ratio of 1:2 (Epi : Mes), and then randomly selected 600 tiles per Epi slide and 300 tiles per Mes slide. However, for slide-wise validation, all the tumor tiles were used. After getting the classification of all tumor tiles of a given slide, the mean prediction value of all these tiles was regarded as the slide’s final prediction result.

### Neural Network and Transfer Learning on Inception V3

Both the tumor detection and EMT subtype detection models were based on Inception V3 and trained using transfer learning. All the convolutional neural networks were pretrained on the ImageNet (www.image-net.org) database. Only the last softmax layer was changed and retrained by our dataset. We used Stochastic gradient descent (SGD) optimization with a learning rate of 0.01 for the classification model. All codes were implemented in Python 3.7 and ran on desktop workstations with Nvidia graphics-processing units (GPUs; NVIDIA 2080). Performance was scored using accuracy, AUC, specificity, and sensitivity.

### Immune Gene Signature Analysis

Immune gene signatures were derived from previously published work (13). RSEM upper quartile normalized, log2 transformed, and mean centered RNA-seq data were matched to predefined immune gene signature clusters *via* Entrez IDs. Each gene signature was included in **Supplementary Table S2**. Differential expression for each gene signature was analyzed between subtypes *via* one-way ANOVA. These P values were adjusted for multiple tests using the Benjamini-Hochberg procedure. For hazard ratio forest plots, a univariate Cox proportional hazards (CoxPH) model was used with signature/clinical variable as a continuous variable compared to patient overall survival.

### CIBERSORT

CIBERSORT is an analytical tool that accurately quantifies the relative levels of distinct immune cell types within a complex gene expression mixture to characterize and quantify each immune cell subtype. Here, we applied the original CIBERSORT gene signature file, LM22, which defined 24 immune cell subtypes, and analyzed datasets from ccRCC. The data were normalized using the cubic spline algorithm. All samples were analyzed for immune cell profiles by CIBERSORT, the number of permutations being set to 100. Twenty-two immune cell types together with CIBERSORT metrics as Pearson correlation coefficient, CIBERSORT *P* value and root mean squared error (RMSE) were quantified for each sample. CIBERSORT *P* value reflects the statistical significance of the deconvolution results across all cell subsets and was useful for filtering out deconvolution with less significant fitting accuracy (https://cibersort.stanford.edu). Immune cell profile was calculated for each sample, and mean values were calculated.

### ESTIMATE

The ESTIMATE algorithm was applied to the normalized expression matrix for estimating the stromal and immune scores for each clear cell renal cell carcinoma sample. Access to the deidentified linked dataset was obtained from TCGA in accordance with the database policy. For analyses of deidentified data from TCGA database, Institutional Review Board approval and informed consent were not required.

### Quantification and Statistical Analysis

Student T test, Wilcoxon test, and Kruskal–Wallis test were utilized to compare continuous variables and ordered categorical variables, such as mutation load, neoantigen load, HRD score,

CTAs number, and ITH. Permutation test was conducted in the comparison of gene mutation frequencies among clusters. Correlation matrices were created with Pearson or Spearman correlation. Survival analysis was performed using the Kaplan–Meier method, and the survival of the clusters was compared using the log-rank test. For all analyses, significance was determined as a *P* value < 0.05 and corrected for multiple testing where specified. Univariate analysis was performed unless otherwise specified. Survival analyses were performed using GraphPad Prisma (GraphPad Software, Inc.) or by individually specified methodologies.

### Data Availability

Data are available from the authors upon request but may require data transfer agreements. No personalized health information will be shared.

### Code Availability

The code used during the current study are available from the corresponding author on reasonable request.

## Results

### Discovery of EMT-Related Gene Signature in Patients With ccRCC

**Figure 1:**
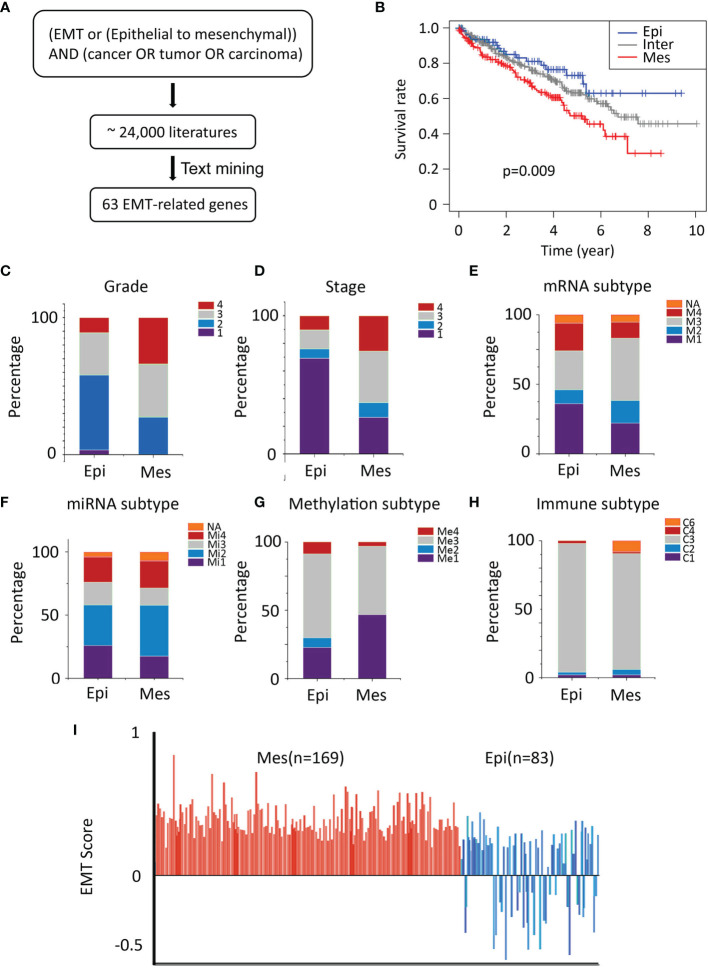
Characteristics of TCGA cohort. **(A)** Text-mining literature abstracts associated with EMT from the PubMed database identified 63 EMT-related genes. **(B)** Overall survival analysis for sample clusters. **(C, D)** General TNM and grade characteristics of patients of the two clusters (left and middle). **(E–G)** Significant differences in mRNA-based, miRNA-based, and methylation classification were identified for both the Epi and Mes clusters. **(H)** The proportion of samples belonging to each immune subtype in two clusters. **(I)** Plot of EMT scores (mean ± SEM; y-axis) of in samples. EMT score nearer to +1.0 is more mesenchymal-like (Mes), whereas EMT score nearer to -1.0 is more epithelial-like (Epi), Epi, blue; Mes, red.

An EMT-related gene signature was developed and consisted of 63 genes (**Supplementary Table S1**) and the flowchart of strategy to identify EMT-related biomarkers in ccRCC was shown in **Figure 1A**. Two distinct subtypes of ccRCC, mesenchymal (Mes) and epithelial (Epi), were identified using hierarchical clustering of gene expression data. The Kaplan-Meier plots showed that the patients in the Mes subtype showed worse overall survival than those in the Epi and Intermediate subtypes (*P* = 0.009; **Figure 1B**).

### Validation of the EMT‐Related Molecular Subtype

The independent cohort (GSE150404) was used to validate the accuracy of the EMT gene signature (*P* < 0.0001; **Supplementary Figure S1**). Furthermore, higher grade (grade 3/grade 4) and stage (stage III/stage IV) samples were in the Mes group (**Figures 1C, D**). Univariate and multivariate analyses were performed and EMT gene signature was significantly associated with outcome in the multivariate analysis (**Table 1**).

**Table 1:** Univariate and multivariate Cox proportional hazard regression analysis of overall survival in the cohorts (n = 252).

Variables	Univariate		Multivariate	
	HR (95% CI)	*P*-value	HR (95% CI)	*P*-value
EMT signature	1.71 (1.27-2.31)	1.66e-09*	1.22 (1.02-1.54)	7.95e-06*
Age	1.04 (1.02-1.06)	0.00026*	1.03 (1.01-1.05)	0.00091*
Gender	1.14 (0.70-1.83)	0.603	0.904 (0.54-1.5)	0.696
Grade	2.57 (1.90-3.47)	8.13e-10*	1.60 (1.12-2.30)	0.01*
Stage	2.01 (1.65-2.46)	8.56e-12*	1.56 (1.12-2.17)	0.0089*
Metastasis	2.89 (1.96-4.25)	6.87e-08*	0.97 (0.5-1.93)	0.97
Lymph node	3.68 (1.69-8.04)	0.001*	1.86 (0.82-4.23)	0.14

*P < 0.05.

According to TCGA Research Network article, unsupervised clustering methods identified subsets in mRNA (m1-m4) and miRNA (mi1-mi4) expression datasets. In the mRNA cluster, m3 accounted for roughly 50% of samples in the Mes classification and was associated with the worst survival outcome (**Figure 1E**). Survival differences were also evident in miRNA-based subtypes (**Figure 1F**). DNA methylation (me1-me3) expression datasets were detected (**Figure 1G**), and almost one-half of the Mes group was me1 type and associated with significantly poorer survival.

Thorsson et al. identified six immune signature sets (C1-C6) associated with overall survival and progression-free interval (14). C3 had the best prognosis and was enriched in most ccRCC, the more mixed-signature subtypes, C4 and C6, had the least favorable outcome. In our model, compared with Epi, Mes had the most significant proportion of C4+C6 (**Figure 1H**).

Further, we quantified the EMT levels by calculating EMT scores described by Tan et al (15). Positive EMT scores corresponded to the mesenchymal phenotype, whereas negative scores reflected the epithelial phenotype. In general, All Mes subtype was characterized by positive EMT scores corresponding to their phenotype. In contrast, Epi phenotype had intermediate and low EMT scores (**Figure 1I**), reflecting their partial EMT and complete epithelial states. These results demonstrated the robustness of the prognostic value of EMT signature.

### Deep Learning Can Predict EMT Status Directly From Histopathology Images

Recently, deep learning has overmatched humans in some medical data processing, especially the ability to predict some genetic information efficiently and economically using histopathology images (16, 17). To facilitate applying the EMT gene signature, we investigated whether deep learning could directly predict EMT status which according to our molecular subtype from H&E-stained slides.

**Figure 2:**
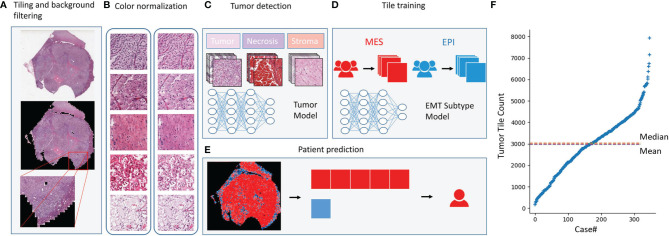
Deep learning model. Deep learning model. **(A)**The whole-slide images were tiled into 256×256-pixel tiles and dropped the tiles with low amount of information while tiling. **(B)** Tiles before and after color normalization. **(C)** The convolutional neural network was trained as a tumor detector at tile level. **(D)** The model training for EMT subtype classification at tile level. **(E)** EMT subtype prediction at patient level. **(F)** Distribution of the number of tiles generated per case.

**Figure 3:**
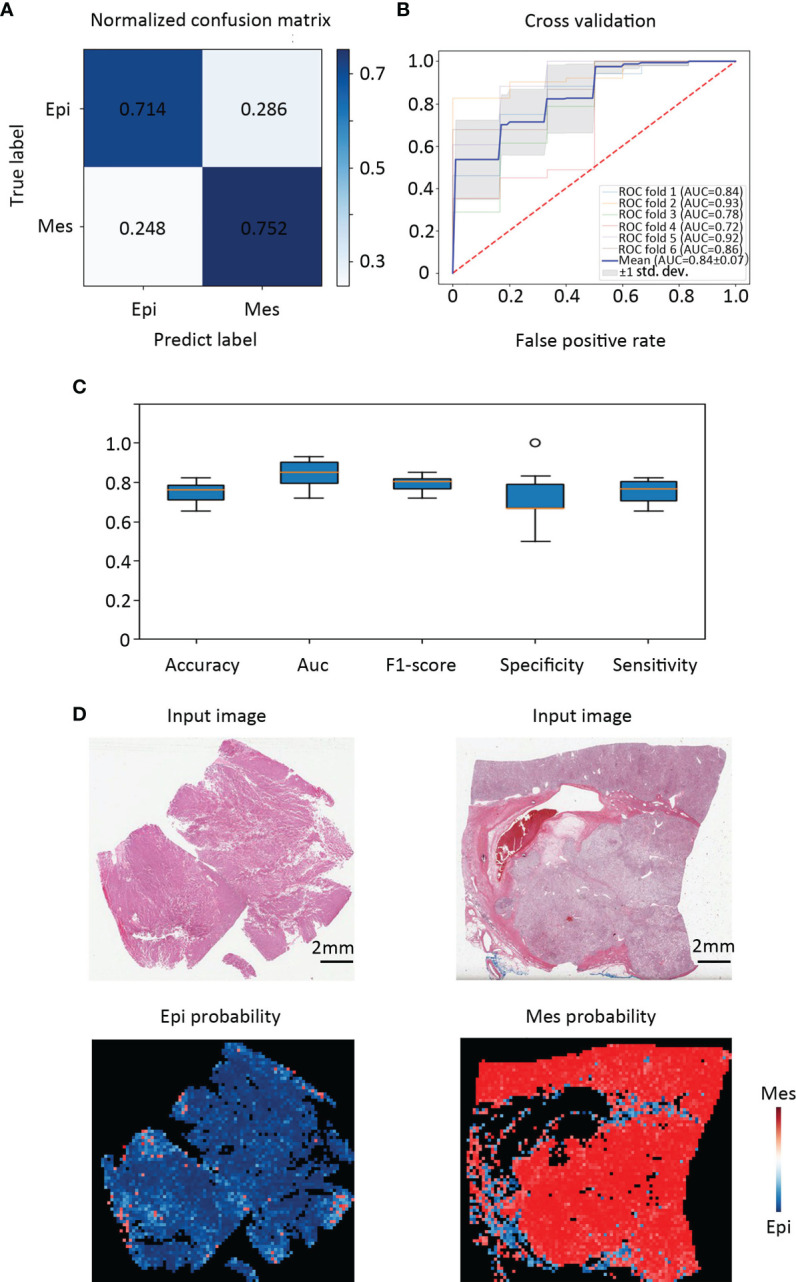
Classification performance in an external validation set and class activation maps for morphological features. **(A)** Confusion matrix of the classification results of the validation patients. **(B)** Training and validation on TCGA cohort were performed using six-fold repeated validation with a 90% (training) to 10% (validation) stratified, random split for each fold. **(C)** Classifier performance was assessed including accuracy, F1 score, AUC, specificity, and sensitivity separately for each EMT molecular subtype. **(D)** Tissue slides of patients with Epi and Mes tumors in TCGA test set show the spatial patterns of predicted MSI score. The top panel shows the H&E input image. Corresponding predicted Epi and Mes map for the image shown in the bottom panel. Class activation maps for morphological features.

In this study, we used 346 whole-slide images from TCGA and labeled them with the EMT types which according to our gene signature (**Figure 2**). Before classifying the EMT status, 3000 tiles of tumor, necrosis, and stroma were selected and labeled by a urologist and a pathologist. We trained an Inception V3 model to recognize tumor from the other two classes by transfer learning, which yielded an average AUC of 0.99 at the tile level in five independent experiments. Then we used the tumor tiles detected by the model with best performance among the five experiments for EMT subtype classification. For the slide-wise six-fold validation of the EMT subtype, the AUC value of the ROC curve for all classes was 0.84 ( ± 0.07). The mean validation accuracy at slide level was 74.90%, mean specificity was 72.23%, and mean sensitivity was 75.32% (**Figures 3A**–**C**). The predicted EMT status and the classification probability, were aggregated to extract the heatmap for visualization by the tiles’ raw position of the slide (**Figure 3D**).

### Histopathological Features Detection for Stratification

**Figure 4:**
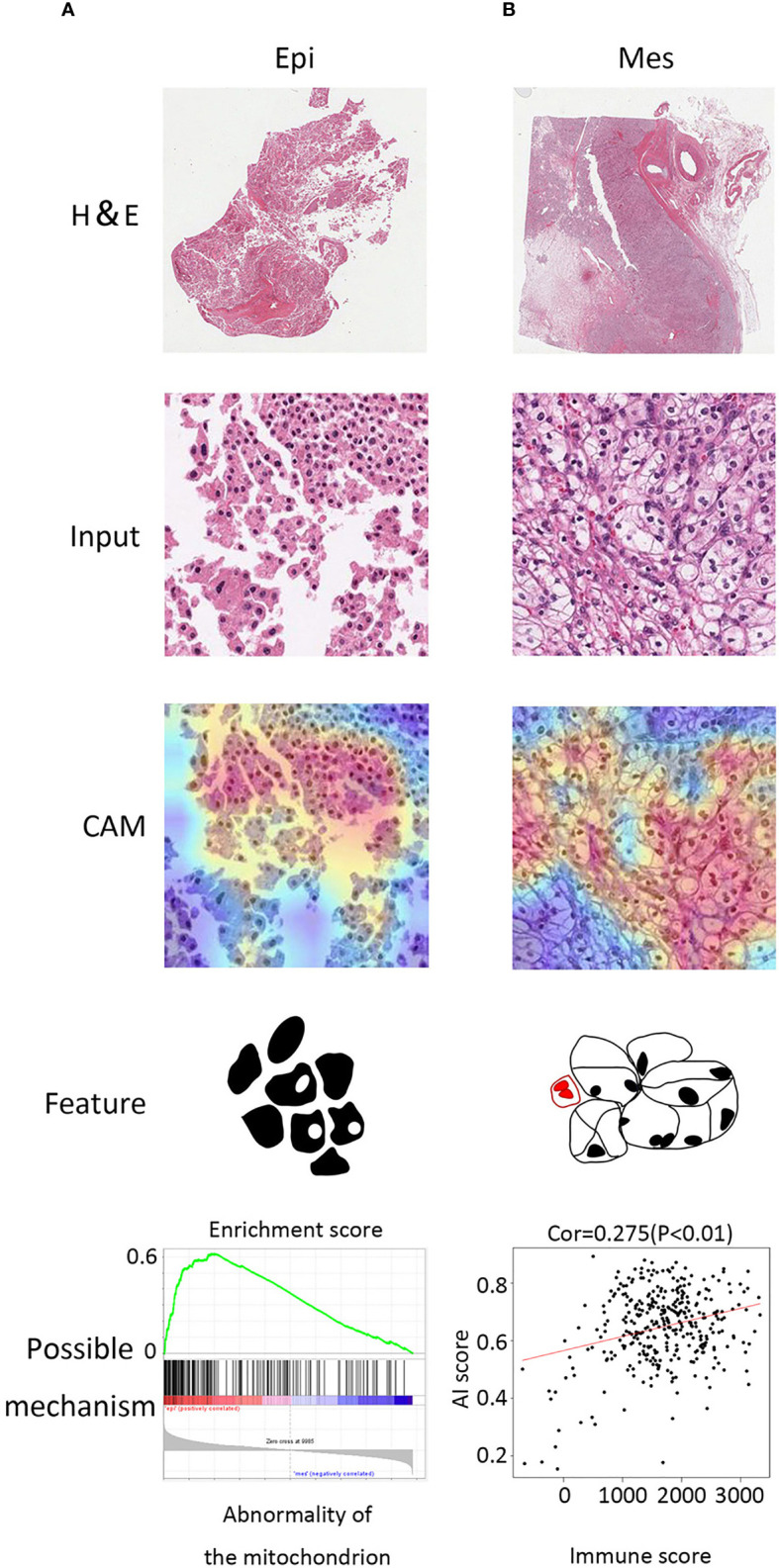
Class activation maps for morphological features. **(A)** Macrograph (top), input image (second from top), CAM (third from top), and corresponding sketch maps of predominant morphological feature (bottom) for EMT subtypes (from left to right: Epi and Mes). Epi region is a granular eosinophilic cytoplasm area with a looser arrangement, big cell gap, nucleoli absent. Mes region contained mostly tumor cells with empty cytoplasm and mixed with immune cells. **(B)** GSEA plot of abnormality of the mitochondrion in Epi cluster (left) and positive correlation between AI score and immune score (estimated by the ESTIMATE algorithm).

To detect which histopathological features were the most relevant in our algorithm to identify molecular subtypes, we used class activation mapping (CAM) (18). For the Epi subtype, these regions had a looser arrangement, big cell gap, nucleoli absent or inconspicuous which were mainly in pink, and granular eosinophilic cytoplasm areas (**Figure 4A**). Several studies suggested that granular eosinophilic cytoplasm reflects the presence of abundant mitochondria, which are required to supply energy for acid secretion (19, 20). For the Mes subtype, the regions were densely packed, surrounded by arborizing vasculature, the large multinucleate cells with empty cytoplasm (different in shape and size), and were often surrounded by abundant immune infiltration (**Figure 4B**).

We further investigated the molecular alterations that could be correlated with the histopathological features. Notably, the genes located in the Mes subtype were annotated to various immune associated pathways and biologic processes (**Supplementary Figure S2**). In addition, we noticed that Mes group exhibited higher artificial intelligence (AI) score (> 0.5 Mes possibility; < 0.5 Epi possibility) and was positively correlated with immune infiltration (Spearman correlation = 0.275, *P* < 0.001, **Figure 4B**). Moreover, gene set enrichment analysis (GSEA) validated that abnormality of the mitochondrion and varieties of metabolic process was enriched in the Epi cluster, which was in accordance with the detection above (**Figure 4A**).

Our tests showed promising results on tumor EMT classification from sections that could be predicted from H&E images. Furthermore, we analyzed the connection between DNA and RNA-level alternations and histopathological features to explore the clinical relevance of the classification.

### Methylation and Metabolism

**Figure 5:**
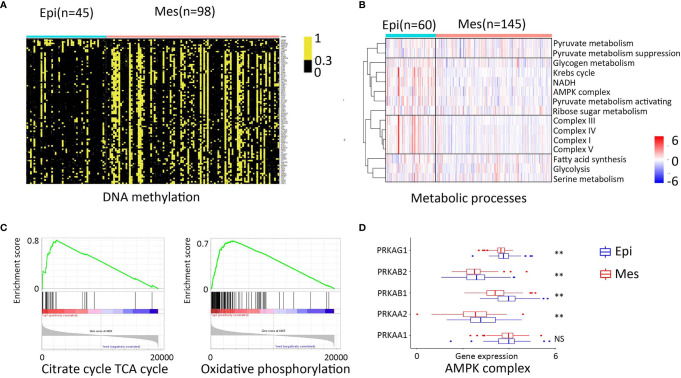
Methylation and metabolism patterns. **(A)** Heatmap representation of the clustering of 89 highly variable DNA methylation probes. A methylation β-value R greater than 0.3 was considered hypermethylated. **(B)** Heatmap representation of the comparison of mRNA expression signatures for the selected metabolic processes between the different EMT subtypes. **(C)** GSEA plot of citrate cycle TCA cycle and oxidative phosphorylation. **(D)** AMPK complex gene expression (**, 0.001 < P < 0.01; ns, P > 0.05).

Heatmapping demonstrated clustering of 89 highly variable DNA methylation markers that were hypermethylated in the tumor. A β-value R greater than 0.3 was considered hypermethylated, and the markers were associated with significantly poorer survival (13). The methylation Mes subtype had a pronounced increase in hypermethylation across the selected genome (**Figure 5A**).

Evaluation of metabolic differences was performed by 15 major metabolic processes. Expression of the Krebs cycle and the electron transport chain genes (complex I – complex IV) provided a clear distinction between the subtypes (**Figure 5B**).

AMP-activated protein kinase (AMPK) acts as an intracellular energy sensor and was significantly lower in Mes subtype compared to Epi subtype (*P* < 0.01; **Figure 5B**), which negatively regulates fatty acid synthesis and positively regulates mitochondria production (21). The metabolic shift identified in the Mes group is reported to contribute to the Warburg metabolic phenotype, further enhanced malignancy, immune protection of cancer cells (22). Furthermore, the GSEA and AMPK complex genes expression validated some of these results (**Figures 5C, D**).

### Mutation and Immune Signature Analysis

**Figure 6:**
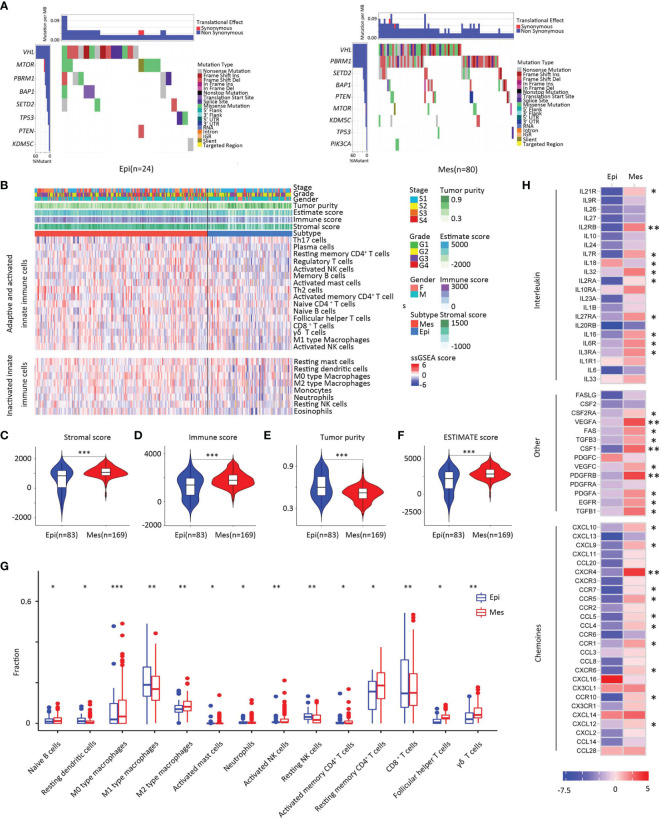
Somatic alterations in EMT subtypes. (A) Upper heat map, mutation events per sample; lower heat map, mutation types, and events (left, Epi group; right, Mes group). **(B)** Landscape of the microenvironment phenotypes in EMT subtypes, K-means clustering of microenvironment phenotypes based on the estimated numbers of 24 microenvironment cell subsets calculated by ssGSEA (Epi type, blue; Mes type red). Tumor stage (stage I, light blue; stage II yellow; stage III, orange; stage IV, red), grade (grade 1, green; grade 2, yellow; grade 3, purple; grade 4, red), gender (male, blue; female, pink), and scores estimated by the ESTIMATE algorithm. **(C–F)** Scores of stromal, immune, tumor purity, and ESTIMATE between clusters. In the violin plots, the mean values are plotted as dots, and the boxplot is drawn inside of the violin plot. **(G)** Boxplot plot for comparison of the immune cell fraction difference between Epi and Mes tissues. Fractions of each immune cell type were compared by means of a two-sided Mann–Whitney U test. Blue color indicates Epi type and red indicates Mes type. **(H)** Log2-fold change in mRNA expression in the tumor tissues of chemokines, interleukins, interferons, and other important cytokines and their receptors for each cluster. Molecules with significantly differential expression between the tumor site and the paired normal site (P < 0.01) were illustrated. (***, P < 0.001; **, 0.001 < P < 0.01; *, 0.01 < P < 0.05; ns, P > 0.05).

The pattern of somatic alterations was determined from the analysis of 104 samples (24 Epi and 80 Mes). Based on previous study methodologies, we identified the eight most significantly mutated genes (*P* < 0.00001) (23). As shown in **Figures 6A, B**, patients with PBRM1 mutation tended to be in the Mes subtype, while the Epi subtype had higher mTOR mutant rates (*P* < 0.05). PBRM1 encodes the bromodomain-containing protein BAF180, a subtype of the switch/sucrose non-fermentable (SWI/SNF) chromatin remodeling complex (24). Importantly, retrospective data have shown that patients with metastatic ccRCC harboring truncating mutations in PBRM1 experienced increased benefit from immune checkpoint therapy (25, 26). Moreover, the Epi subtype tended to have a better response to the mTOR inhibitor.

To further investigate the relationship between the EMT-associated subtype and the immune, we first established a microenvironment phenotype calculated by ssGSEA (**Supplementary Table S2**). Mes subtype was characterized by high innate and adaptive immune cells infiltration (**Figure 6C**). The ESTIMATE algorithm showed that the Mes subtype had the highest immune and stromal scores for the analyzed cohort (*P* < 0.01; **Figures 6D**–**G**).

To identify whether different clusters of EMT had distinct tumor extrinsic immune escape mechanisms (27), we focused on the adaptive immune cells in ccRCC (**Figure 6H**). Analysis revealed that the fraction of naïve B cells was higher in Mes subtype tissue than in Epi tissue. The three main T cell subpopulations in tissue (CD4+ memory resting T cells, CD8+ T cells, and follicular helper T cells) were increased in Mes compared to Epi. The results also showed that innate immune components, neutrophils, M2 macrophages and resting NK fractions increased. In general, Mes had abundant active innate and adaptive immune cells and immunosuppressive cells.

Consistent with these results (**Figure 6I**), Mes had a higher expression of chemokines, including CCL4, CXCL9, and CXCL10, which have been proven to attract dendritic cells and CD8 T cells (28). Overall, the increase of chemokines might contribute to the extrinsic immune escape of the Mes clusters.

**Figure 7:**
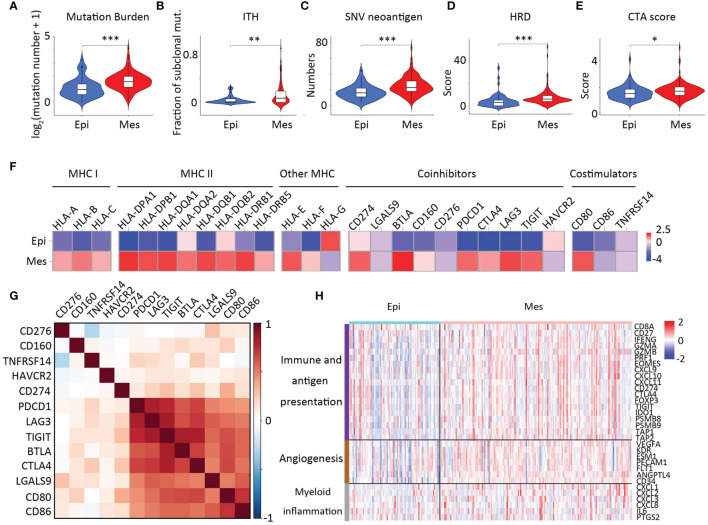
Intrinsic immune escape mechanisms of EMT subtypes. Comparison of mutation burden **(A)**, ITH **(B)**, SNV neoantigen **(C)**, HRD scores **(D)**, and CTA numbers **(E)** among the two clusters. In the violin plots, the mean values are plotted as red dots, and the boxplot was drawn inside the violin plot. **(F)** Comparison of the log2-fold changes in mRNA expression at the tumor sites of the MHC molecules, costimulators, and coinhibitors for each cluster. For costimulators, only molecules having significantly differential expression between two clusters (except CD274 and CD276, P < 0.01) were illustrated. **(G)** Correlations between expression of immune checkpoint molecules. **(H)** Heatmap showing expression of genes related to angiogenesis (brown), immune and antigen presentation (purple), and myeloid inflammation (gray) were z-score transformed before visualization.

We further investigated the two aspects of intrinsic immune escape: tumor immunogenicity and immune checkpoint molecule expression (29). We first compared the factors that were the primary source of tumor antigens: mutation load, neoantigen load, CTA (cancer-testis antigen) level, HRD (homologous recombination deficiency), ITH (intratumoral heterogeneity), and tumor antigen-presenting capability. In general, the difference in the tumor antigen burden among the clusters was significant between the two groups (**Figures 7A**–**E**). Considering that high tumor mutation burden (TMB) correlates with a greater probability of displaying tumor neoantigens, it is rational to hypothesize that the tumors with Mes subtype are more likely to respond to immune checkpoint inhibitors (ICIs) as this greater mutation load.

Therefore, we referred to a database of immune checkpoint molecules among clusters (30) (**Figure 7F**). In many cancers, the PD-1 pathway is involved in tumor evasion from immune activity (31). PD-1 (PDCD1) and CTLA-4 (CTLA4) levels were significantly elevated in the Mes subtype as compared with those in the Epi subtype (p < 0.01); Furthermore, we demonstrated that PD-1, CTLA4, LAG3, TIGIT, CD80, and CD86 and the expression of most checkpoint molecules were positively correlated (**Figure 7G**). In addition, analysis of overall survival of CTLA4 was positively correlated with poor patient survival (**Supplementary Figure S3**).

To further test the clinical relevance, a heatmap of genes previously defined and representing angiogenesis and immune biology was investigated (**Figure 7H**) **(** 32). Our data suggested the association of Mes subtype with improved response to antiangiogenic drugs. We further conducted confirmatory studies to evaluate molecular subtypes by deep learning method in 34 stage IV patients enrolled in the study from 2 January 2020 to 2 June 2021. Patients received more than one dose of pazopanib. In our cohort, only four patients in the pazopanib arm were identified as Epi term, the average score of the remaining patients was 0.72 (95% CI, 0.54–0.83) for Mes tiles and 0.28 (95% CI, 0.16–0.40) for Epi tiles (**Supplementary Table S3**). In Mes-type patients, the objective response rates were 47% (10% complete response; 37% partial response); no Epi-type patients benefitted from the pazopanib. Of note, we observed a trend for improved efficacy (objective response rates) with Mes subtype, indicating that our approach can be beneficial to targeted cancer therapy.

## Discussion

Although pathology divides tumors into distinct ccRCC grades, it is limited in its ability to provide an in-depth analysis (33). Using deep learning methods, our study revealed EMT subtypes and their clinical significance.

In the present study, a robust EMT gene signature clinically significant to patients with ccRCC was developed. Moreover, further analysis demonstrated that Mes and Epi ccRCC subtypes were characterized by distinctive mutations, chromosomal copy number alterations, and mRNA, miRNA, methylation expression, and metabolic patterns. The Mes subtype metabolic alterations were in accordance with the histopathological feature detection. These findings suggested that treatment with demethylating agents and targeting the metabolic pathway could benefit patients with Mes phenotype.

The role of the features in determining the therapeutic responsiveness of ccRCC will be important in future therapeutic planning (34, 35). Studies have reported that EMT are related to immunosuppressive cytokines in several cancer types (36). It has also been reported that EMT can induce PD-L1 expression in non-small cell lung cancer (37). Our study has important implications for clinical translations. First, our results might facilitate the selection of suitable patients for ICIs. We revealed that the Mes cluster demonstrated truncating mutations in PBRM1, and high expression of immune checkpoint molecules might lead to the immune escape of this cluster. Further we suggested that patients in the Mes subtype might respond better to ICIs combined with antiangiogenic therapy. Notably, the CheckMate 9ER trial demonstrated improved progression-free survival and overall survival benefits with the combination of cabozantinib plus nivolumab (38). A Keynote-426 update demonstrated ongoing overall survival benefits of pembrolizumab plus axitinib in the intention-to-treat population (38). These combination therapies are recommended as first-line treatment for advanced kidney cancer. Therefore, our data suggest that EMT signature-based biomarkers may be valuable for identifying patients who can benefit from immune checkpoint blockade and antiangiogenesis agents.

However, the EMT molecular subtype analysis in ccRCC specimens is far from being implemented in routine pathology due to the high costs. We demonstrated how the EMT gene signature could further benefit from deep learning by presenting a strategy based on convolutional neural networks. The development of inexpensive and more powerful technologies has made the training of larger and more complex neural networks possible.

## Conclusions

In conclusion, our analysis showed that the EMT molecular subtype of ccRCC specimens *via* deep-learning convolutional neural networks could be an extremely useful tool for patient-tailored therapy strategies.

## Data Availability Statement

The original contributions presented in the study are included in the article/**Supplementary Material**. Further inquiries can be directed to the corresponding authors.

## Ethics Statement

The studies involving human participants were reviewed and approved by Chinese Clinical Trial Registry. The patients/participants provided their written informed consent to participate in this study.

## Author Contributions

DY had full access to all the data in the study and takes responsibility for the integrity of the data and the accuracy of the data analysis. Study concept and design: QC. Acquisition of data: YK, SW, XZ, and HW. Analysis and interpretation of data: LC, QC, YK, SW, XZ, HW, and WL. Drafting of the manuscript: QC and LC. Critical revision of the manuscript for important intellectual content: DY and LC. Statistical analysis: QC and WL. Administrative, technical, or material support: YK. Supervision: HW. All authors contributed to the article and approved the submitted version.

## Conflict of Interest

The authors declare that the research was conducted in the absence of any commercial or financial relationships that could be construed as a potential conflict of interest.

The reviewer AG declared a past co-authorship with one of the authors LC to the handling editor.

## Publisher’s Note

All claims expressed in this article are solely those of the authors and do not necessarily represent those of their affiliated organizations, or those of the publisher, the editors and the reviewers. Any product that may be evaluated in this article, or claim that may be made by its manufacturer, is not guaranteed or endorsed by the publisher.

## Supplementary Material

Supplementary Figure 1 Kaplan-Meier plots of OS of the cohort.

Supplementary Figure 2 GSEA plot of cytokine recceptor interaction, JAK-STAT pathway, chemokine signaling pathway, regulation of alpha beta T cell activation and differentiation in Mes cluster.

Supplementary Figure 3 Kaplan-Meier plots of OS of the different expression levels of immune checkpoint molecules.

## Abbreviations

TCGA, The Cancer Genome Atlas; EMT, Epithelial–mesenchymal transition; ccRCC, clear cell renal cell carcinoma; Mes, mesenchymal; Epi, epithelial; CAM, class activation mapping; AMPK, AMP-activated protein kinase; CTA, cancer-testis antigen; HRD, homologous recombination deficiency; ITH, intratumoral heterogeneity; TMB, tumor mutation burden; ICIs, immune checkpoint inhibitors.
