# Supplementary figures and images for: Deep Learning–Based Classification of Epithelial–Mesenchymal Transition for Predicting Response to Therapy in Clear Cell Renal Cell Carcinoma
Source: Front Oncol. 2022 Jan 24;11:782515. doi: 10.3389/fonc.2021.782515 (PMC8819137; doi:10.3389/fonc.2021.782515)

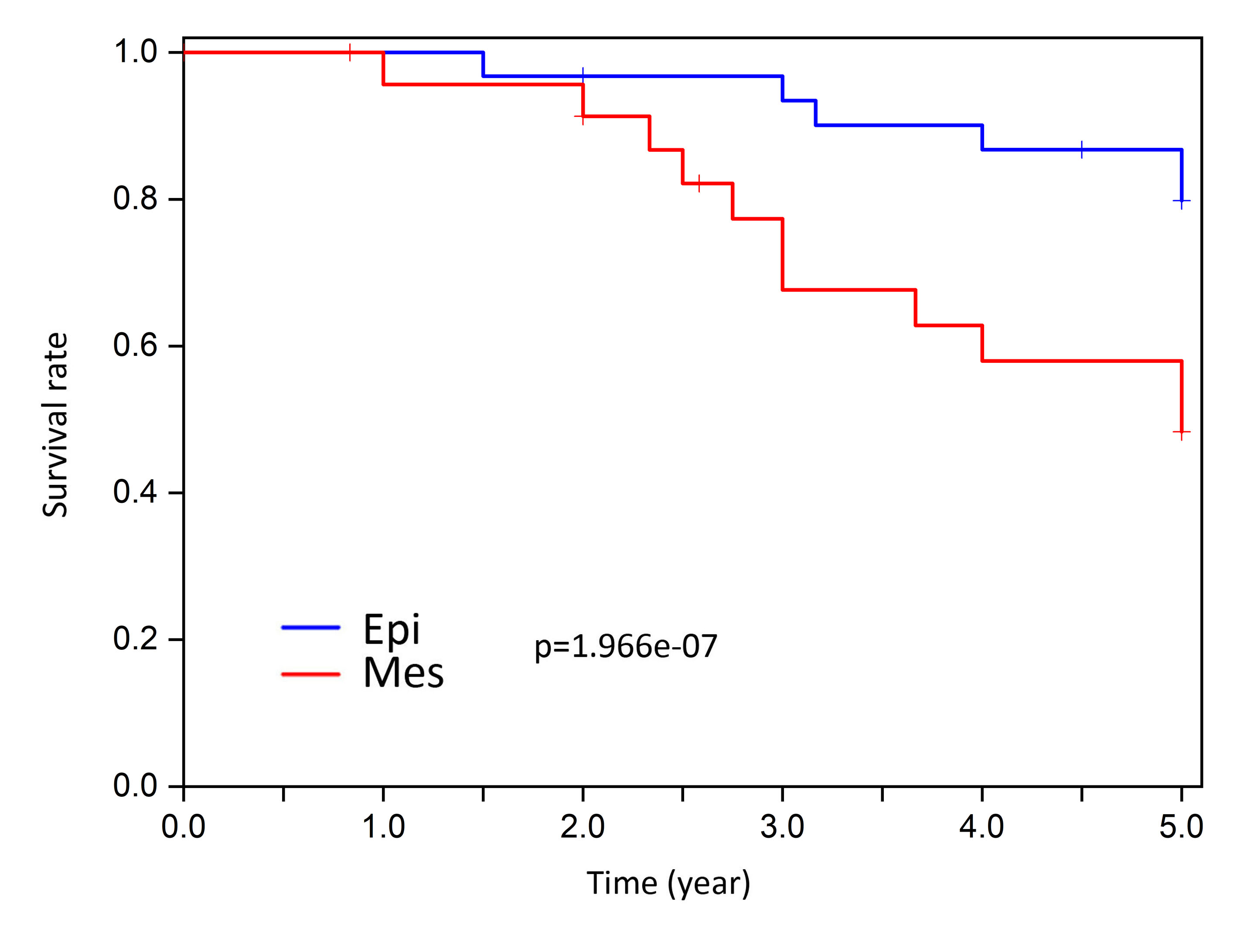

Supplement: Supplementary Figure 1 — Kaplan-Meier plots of OS of the cohort. [file Image_1.jpeg]

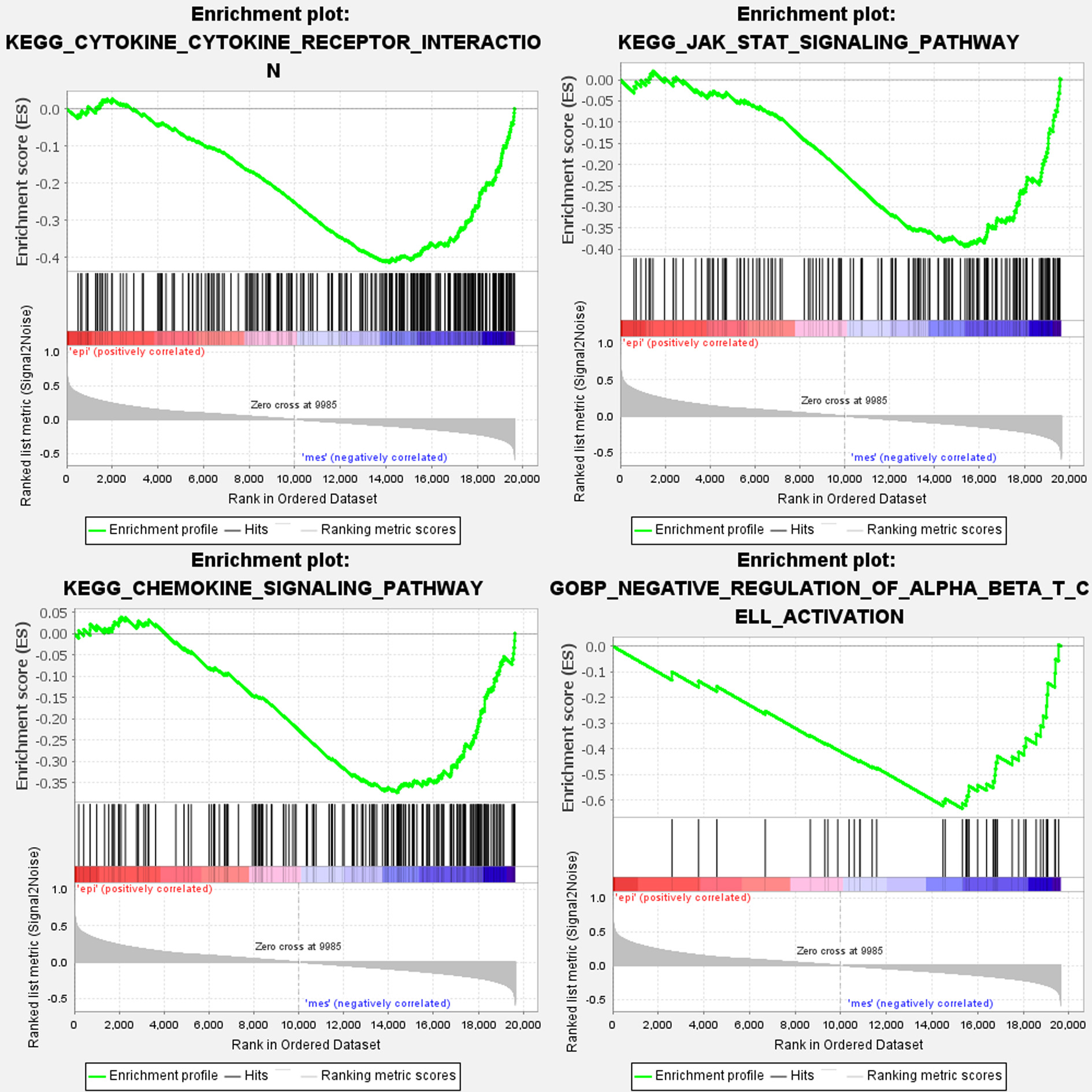

Supplement: Supplementary Figure 2 — GSEA plot of cytokine recceptor interaction, JAK-STAT pathway, chemokine signaling pathway, regulation of alpha beta T cell activation and differentiation in Mes cluster. [file Image_2.jpeg]

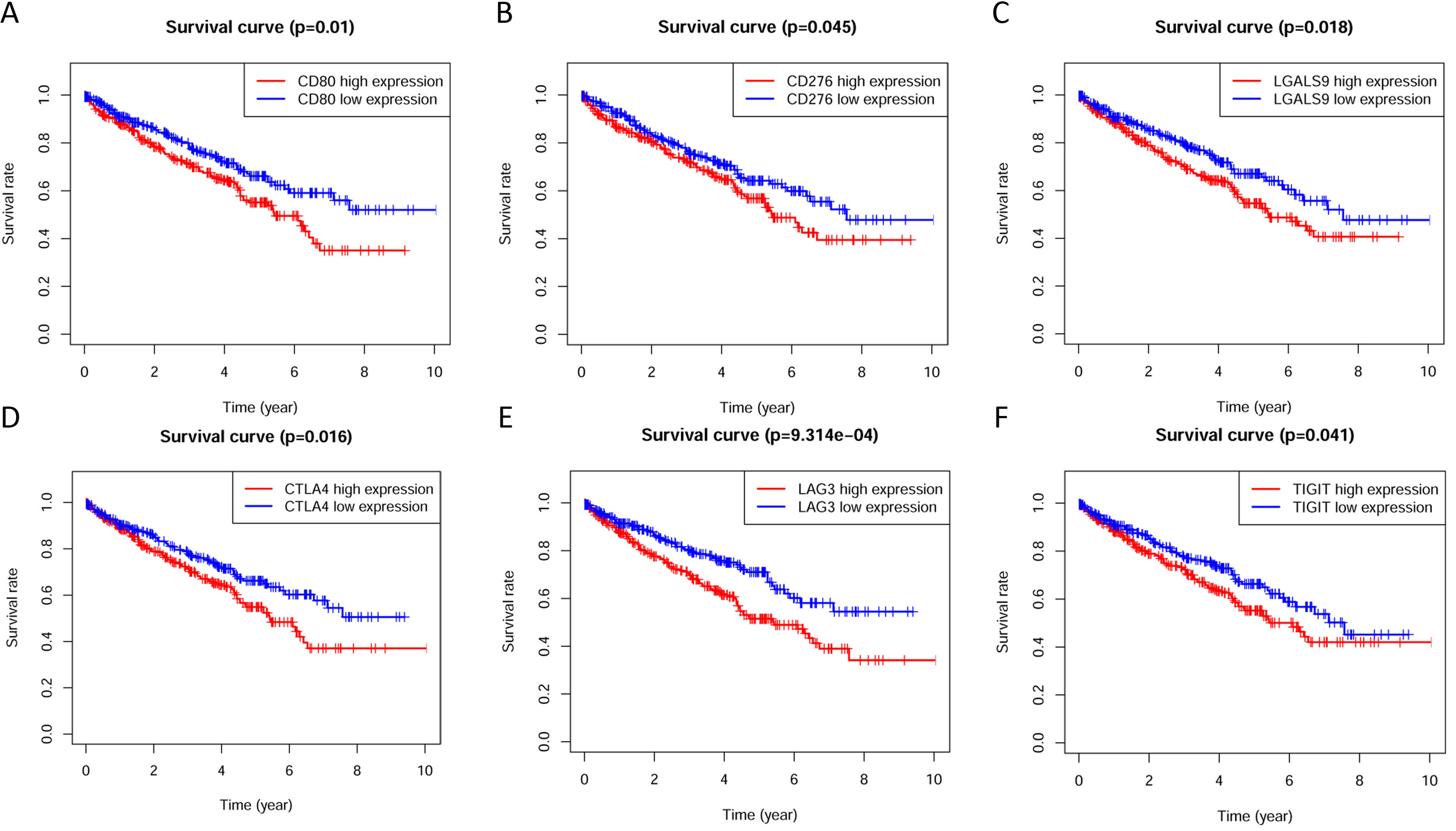

Supplement: Supplementary Figure 3 — Kaplan-Meier plots of OS of the different expression levels of immune checkpoint molecules. [file Image_3.jpeg]
